# External Validation of a Prognostic Model Predicting Metastatic Castration-Resistant Prostate Cancer Survival in Patients Receiving Post-Docetaxel Second-Line Chemotherapy

**DOI:** 10.31662/jmaj.2021-0198

**Published:** 2022-03-04

**Authors:** Leandro Blas, Masaki Shiota, Motonobu Nakamura, Akira Yokomizo, Toshihisa Tomoda, Naotaka Sakamoto, Narihito Seki, Shuji Hasegawa, Takakazu Yunoki, Masahiko Harano, Kentaro Kuroiwa, Masatoshi Eto

**Affiliations:** 1 Department of Urology, Graduate School of Medical Sciences, Kyushu University, Fukuoka, Japan; 2 Department of Urology, National Hospital Organization Kyushu Cancer Center, Fukuoka, Japan; 3 Department of Urology, Harasanshin Hospital, Fukuoka, Japan; 4 Department of Urology, Oita Prefectural Hospital, Oita, Japan; 5 Department of Urology, National Hospital Organization Kyushu Medical Center, Fukuoka, Japan; 6 Department of Urology, Kyushu Central Hospital, Fukuoka, Japan; 7 Department of Urology, Kitakyushu Municipal Medical Center, Kitakyushu, Japan; 8 Department of Urology, Japanese Red Cross Fukuoka Hospital, Fukuoka, Japan; 9 Department of Urology, JCHO Kyushu Hospital, Kitakyushu, Japan; 10 Department of Urology, Miyazaki Prefectural Miyazaki Hospital, Miyazaki, Japan

**Keywords:** castration-resistant prostate cancer, prognosis, cabazitaxel, docetaxel

## Abstract

**Introduction:**

The Halabi model predicts the overall survival (OS) of patients with metastatic castration-resistant prostate cancer (mCRPC) treated with second-line therapy after docetaxel. We aimed to validate this model externally with an independent cohort, outside the setting of a clinical trial.

**Methods:**

In a multi-institutional study, we included 66 patients treated with cabazitaxel after docetaxel for mCRPC. Patients were stratified according to the two- and three-risk groups of the Halabi nomogram. Kaplan-Meier and Cox proportional hazard analyses were performed to estimate survival and hazard ratios (HRs). The model performance was assessed using receiver operating characteristic curves, and the associated c-index (area under the curve [AUC]).

**Results:**

The median OS in the two-risk groups was 5.06 months in the high-risk group (n=22) and 12.9 months in the low-risk group (n=44, p<0.001). High-risk patients had an HR of 9.50 (95% confidence interval (CI) 4.12-21.6, p<0.001) compared to low-risk patients. For the three-risk groups, the median OS was 6.44 months in the high-risk group (n=15), 5.75 months in the intermediate-risk group (n=11), and 13.7 months in the low-risk group (n=40, p=0.84). Compared to low-risk patients, intermediate-risk patients had an HR of 7.49 (95% CI 3.08-20.4, p<0.001), and high-risk patients had an HR of 8.48 (95% CI, 3.39-21.7, p<0.001). The AUC was 0.72 (95% CI 0.64-0.76) for the two-risk stratification. When comparing different risks, the AUCs were 0.48 (high vs intermediate), 0.66 (high vs low), and 0.65 (intermediate vs low).

**Conclusions:**

The two-risk stratification version but not the three-risk group analysis confirmed the ability of the model to predict survival. These results support the value of the Halabi nomogram in men receiving post-docetaxel second-line chemotherapy for mCRPC.

## Introduction

Worldwide, prostate cancer was the second most frequently diagnosed cancer in men and the fifth cause of cancer mortality in 2020 ^(1)^. The natural course of recurrent or advanced prostate cancer is progression to castration-resistant prostate cancer (CRPC) ^(2), (3), (4)^. Several therapeutic agents that prolong survival in men with metastatic CRPC (mCRPC) have been developed ^(5)^. Cabazitaxel is a novel semisynthetic taxane derivative (such as docetaxel) with activity in docetaxel-resistant cancers. The TROPIC trial compared the efficacy and safety of cabazitaxel plus prednisolone with those of mitoxantrone plus prednisolone in men with mCRPC with progression after docetaxel ^(6)^. Current guidelines recommend treatment with cabazitaxel in patients with mCRPC and progression following docetaxel chemotherapy, among other treatment options, including abiraterone, enzalutamide, and radium-223 ^(7), (8)^. However, the adequate sequencing of these therapies to obtain the greatest benefit has not been well explained. For this purpose, several prognostic models that predict survival in patients undergoing chemotherapy have been developed and validated ^(9), (10), (11), (12)^.

Halabi and colleagues developed a prognostic model to predict overall survival (OS) in patients with mCRPC treated with second-line therapy after docetaxel ^(11)^. They derived this model using data from the TROPIC trial, an open-label, multicenter phase III randomized trial ^(6)^. The trial included 378 men with mCRPC who were treated with docetaxel and 377 men treated with mitoxantrone. The authors externally validated the model with data from the SPARC trial, a randomized, double-blind, placebo-controlled trial ^(13)^, and compared the safety and efficacy of satraplatin and prednisone versus placebo plus prednisone in men with mCRPC previously treated with one cytotoxic regimen. The model had the following nine pre-second-line chemotherapy parameters: (i) Eastern Cooperative Oncology Group performance status of 2, (ii) time to progression on docetaxel <6 months (iii) presence of measurable disease, (iv) presence of visceral disease, (v) pain, (vi) duration of hormonal use, (vii) hemoglobin, (viii) prostate-specific antigen (PSA), and (ix) alkaline phosphatase. This model stratifies patients into two-risk groups (low and high) or three risk groups (low, intermediate, and high). However, the model has not been validated in patients treated outside the setting of a clinical trial. Thus, this study aimed to validate this model externally in an independent cohort.

## Materials and Methods

### Patients

We included patients with mCRPC treated with cabazitaxel chemotherapy between 2014 and 2017 at the following institutions: Kyushu University Hospital (Fukuoka), National Hospital Organization Kyushu Cancer Center (Fukuoka), Harasanshin Hospital (Fukuoka), Oita Prefectural Hospital (Oita), National Hospital Organization Kyushu Medical Center (Fukuoka), Kyushu Central Hospital (Fukuoka), Kitakyushu Municipal Medical Center (Kitakyushu), Japanese Red Cross Fukuoka Hospital (Fukuoka), JCHO Kyushu Hospital (Kitakyushu), and Miyazaki Prefectural Miyazaki Hospital (Miyazaki) ^(3)^. This study was approved by the institutional review board of Kyushu University, approval number 2019-230. Patients with (i) a histopathological diagnosis of carcinoma of the prostate; (ii) age ≥20 years; and (iii) progression despite primary androgen-deprivation therapy were included. Patients who received a single administration of cabazitaxel were excluded. Clinical information at the start of cabazitaxel therapy was collected.

### Treatment

Cabazitaxel (20-25 mg/m^2^) was administered on a 3- or 4-weekly regimen based on the schedule reported by the TROPIC ^(6)^ and PROSELICA trials ^(14)^. Only one patient was treated with 15 mg/m^2^ cabazitaxel. Prednisolone 5 mg was given twice daily simultaneously with medical or surgical castration.

### Measurements

OS was defined as the time from the beginning of treatment with cabazitaxel to the time of death by any cause. Disease progression was defined according to the Prostate Cancer Clinical Trials Working Group ^(15)^. Pain was defined by the daily consumption of narcotic or non-narcotic analgesics for pain derived from prostate cancer. The performance status was determined according to the Eastern Cooperative Oncology Group criteria. The presence of visceral metastases was defined as metastases in the lungs, liver, pancreas, and adrenal gland. Using the online Halabi nomogram, the estimated prognosis of OS was calculated for each patient for the two- and three-risk versions ^(11)^.

### Statistical analyses

Baseline values were expressed as the median and interquartile range (IQR), and the baseline was defined as the date of initial cabazitaxel chemotherapy. Survival analysis was performed using the Kaplan-Meier method with the Rothman 95% confidence interval (CI). The log-rank test and Cox proportional hazards model were used to detect differences in survival between groups. The discrimination of the model was assessed using receiver operating characteristic curves, and the associated c-index (area under the curve [AUC]). The performance was evaluated at 12, 15, 18, and 24 months. All statistical analyses were performed using Stata v.17 (College Station, TX, USA). A p-value <0.05 was considered statistically significant.

## Results

**Figure 1.**
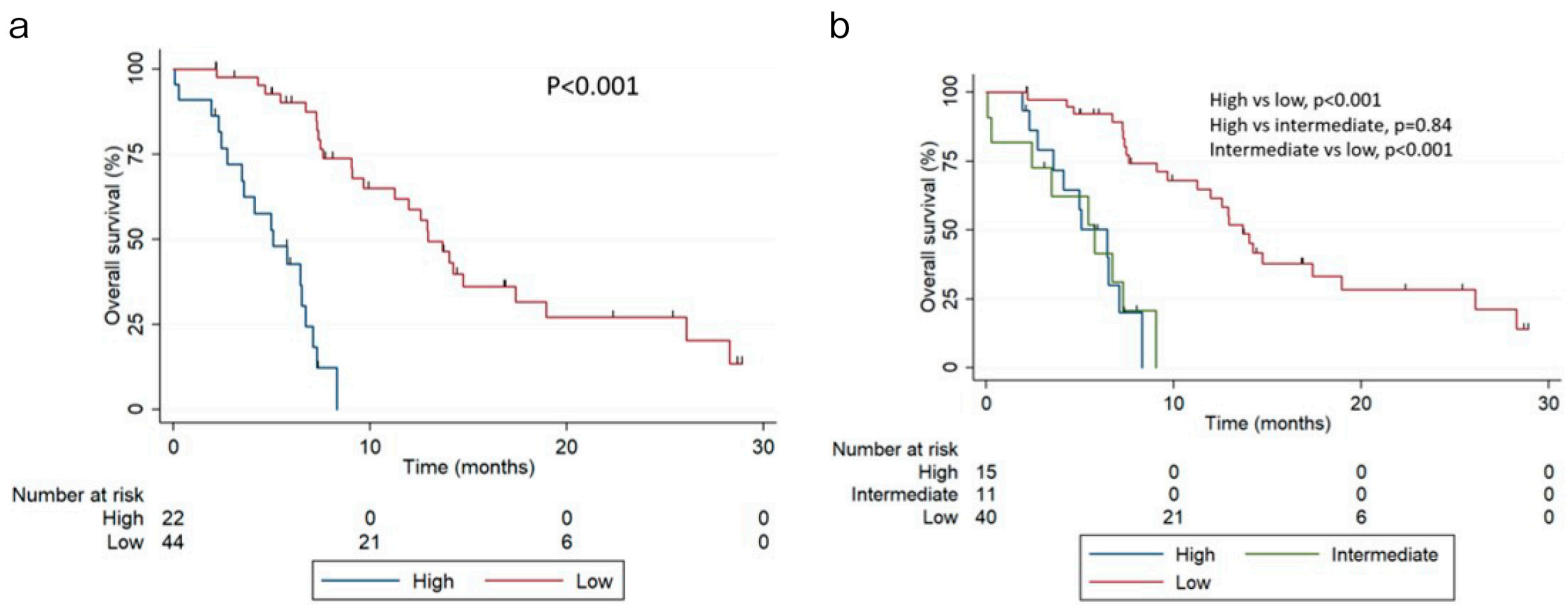
Kaplan-Meier survival curves showing OS stratified by (a) the two-risk group and (b) the three-risk group.

Of the initial 74 patients, after excluding men with incomplete data (n=8), 66 patients were included in the final analysis. The patients’ background characteristics are shown in Table 1. The median follow-up was 7.28 months (IQR 4.93-12.9 months), and the median OS was 9.04 months (95% CI 7.26-12.9 months). When patients were stratified using the two-risk group version, 22 (33.3%) men showed high risk and 44 (66.6%) men showed low risk. OS at 6 months was 42.6% (95% CI 21.5%-62.3%) and 90.1% (95% CI 75.6%-96.2%) for high- and low-risk patients, respectively. The median OS was 5.06 months (95% CI 2.72-6.70 months) and 12.9 months (95% CI 9.66-17.4 months) for high- and low-risk patients, respectively (Figure 1a, p<0.001). In the three-risk group version, 15 (22.7%), 11 (16.7%), and 40 (60.6%) men had high, intermediate, and low risk, respectively. OS at 6 months was 50.2% (95% CI 22.1-72.4), 41.6% (95% CI 13.1-68.4), and 92.1% (95% CI 77.4%-97.4%) for high-, intermediate-, and low-risk patients, respectively. The median OS was 6.44 months (95% CI 2.72-7.11 months), 5.75 months (95% CI 0.2-7.29 months), and 13.7 months (95% CI 9.6-18.9 months) for high-, intermediate-, and low-risk patients, respectively (Figure 1b).

**Table 1.** Patients’ Background Characteristics.

	All patients (n=66)
Median age (IQR), years	73 (67-76)
Biopsy Gleason Score, n (%)	
≤7	11 (17.4)
8	11 (17.4)
≥9	41 (65.2)
NA	3
Prior local therapy, n (%)	
Presence	19 (28.8)
Absence	47 (71.2)
Measurable disease, n (%)	46 (69.7%)
Pain, n (%)	30 (45.4%)
Progression on docetaxel <6 months, n (%)	54 (81.8)
Number of docetaxel cycles, median (IQR)	7 (5-10)
Prior treatment for CRPC, n (%)	
Abiraterone/Enzalutamide	56 (84.5)
Radium-223	4 (6.06)
Performance status	
0	43 (65.1%)
1	15 (22.7%)
2	8 (12.2%)
Median time on hormone treatment (IQR), years	3.54 (2.23-6.74)
Median hemoglobin (IQR), g/dL	12.1 (10.9-12.9)
Median alkaline phosphatase (IQR), IU/L	288 (204-496)
Median PSA (IQR), ng/mL	59.1 (18.1-176.4)
Metastatic sites, n (%)
Bone	58 (87.8)
Lymph node	39 (59.1)
Visceral	18 (27.2%)
Number of cabazitaxel cycles, median (IQR)	5 (3-8)

CRPC, castration-resistant prostate cancer; IQR, interquartile range; PSA, prostate-specific antigen

Compared to low-risk patients, intermediate-risk patients had an HR of 7.49 (95% CI 3.08-20.4, p<0.001), and high-risk patients had an HR of 8.48 (95% CI, 3.39-21.7, p<0.001). In the two-risk group version, high-risk patients had an HR of 9.50 (95% CI 4.12-21.6, p<0.001) compared to low-risk patients. The AUCs were 0.72 (95% CI 0.64-0.76) and 0.69 (95% CI 0.62-0.74) for the two- and three-risk groups, respectively. Table 2 shows the AUC at 12, 15, 18, and 24 months for both risk groups. When comparing different risks, the AUCs were 0.48 (high vs intermediate), 0.66 (high vs low), and 0.65 (intermediate vs low).

**Table 2.** AUC Calculations for Groups at Various Time Intervals, Including the Number of Patients Who Died or Progressed at Each Time Point.

		OS			PFS	
		Two-risk group	Three-risk group		Two-risk group	Three-risk group
Time point (months)	Death, n (%)	AUC (95% CI)	AUC (95% CI)	Progression, n (%)	AUC (95% CI)	AUC (95% CI)
12	33 (50.0)	0.67 (0.59-0.75)	0.64 (0.54-0.73)	43 (76.7)	0.63 (0.54-0.69)	0.65 (0.53-0.73)
15	40 (60.6)	0.70 (0.63-0.76)	0.67 (0.61-0.75)	45 (80.3)	0.62 (0.54-0.69)	0.64 (0.58-0.74)
18	41 (62.1)	0.71 (0.63-0.76)	0.68 (0.62-0.75)	46 (82.1)	0.62 (0.56-0.69)	0.65 (0.59-0.70)
24	42 (63.6)	0.71 (0.64-0.76)	0.69 (0.62-0.76)	47 (83.9)	0.63 (0.56-0.70)	0.65 (0.60-0.71)

AUC, area under the curve; CI, confidence interval; OS, overall survival; PFS, progression-free survival

PSA response information was available in 56 patients. Patients received a median of five cycles of cabazitaxel therapy (IQR 3-8) and had previously received a median of seven cycles of docetaxel therapy (IQR, 5-10). In the entire patient cohort, 40 (60.6%), 11 (16.6%), and 7 (10.6%) patients experienced treatment failure because of disease progression, adverse effects, and patient requests, respectively, and cabazitaxel treatment was completed in the remaining 8 patients (12.1%).

**Figure 2.**
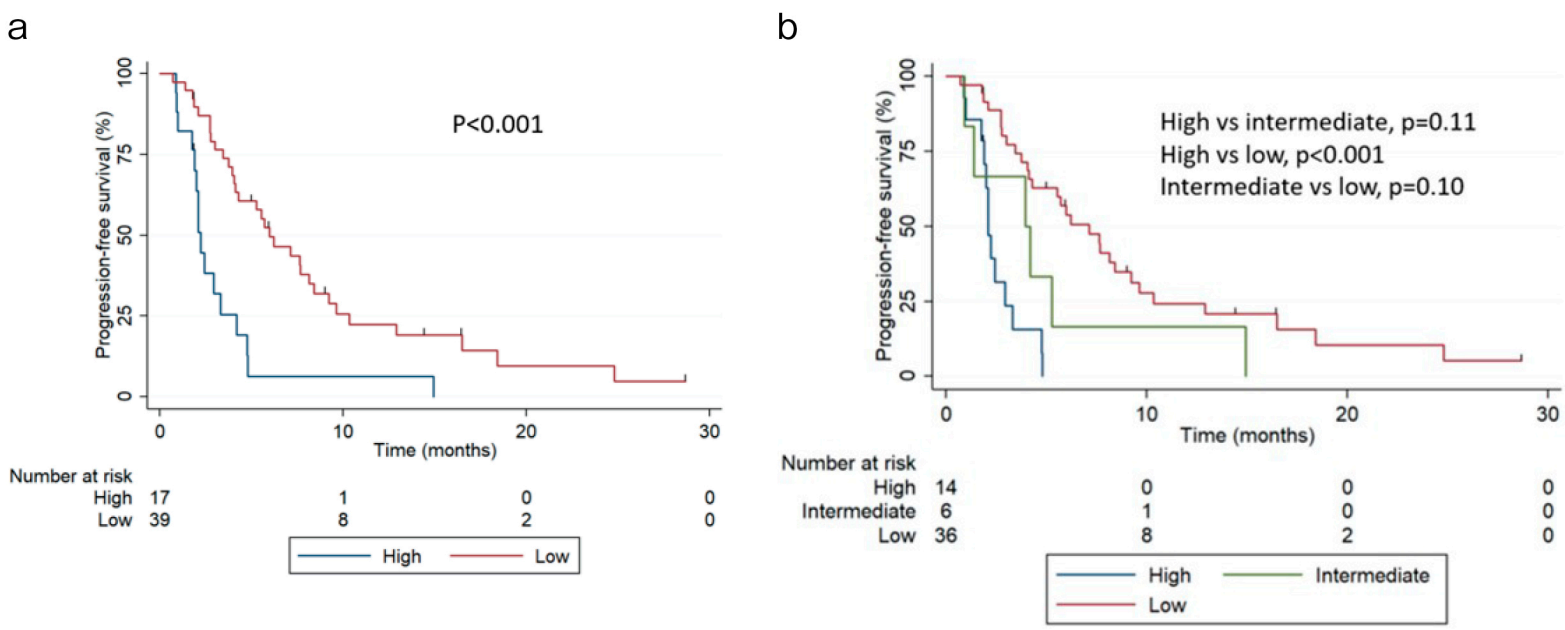
Kaplan-Meier survival curves showing PFS stratified by (a) the two-risk group and (b) the three-risk group.

During the follow-up, 48 patients (85.7%) experienced progression with cabazitaxel. The median progression-free survival (PFS) was 4.27 months (95% CI 2.99-5.98 months). PFS at 6 months was 6.37% (95% CI 0.4%-25.7%) and 49.9% (95% CI 32.7%-64.1%) for the high- and low-risk groups, respectively. The median PFS was 2.2 months (95% CI 1.7-3.2 months) and 5.98 months (95% CI 4.04-8.41 months) for the high- and low-risk groups, respectively (Figure 2a, p<0.001). In the three-risk groups, 14 (25.0%), 6 (10.7%), and 36 (64.3%) men had high, intermediate, and low risk, respectively. PFS at 6 months was 0%, 16.3% (95% CI 0.70%-51.8%), and 53.9% (95% CI 35.8%-68.6%) for the high-, intermediate-, and low-risk groups, respectively. The median PFS was 2.10 months (95% CI 1.7-2.9), 3.94 months (95% CI 1.71-not reached), and 7.1 months (95% CI 4.1-9.2 months) for the high-, intermediate-, and low-risk groups, respectively (Figure 2b). The AUCs were 0.63 (95% CI 0.54-0.69) and 0.65 (95% CI 0.59-0.71) for the two- and three-risk groups models, respectively. When comparing different risks, AUCs were 0.56 (high vs intermediate), 0.65 (high vs low), and 0.55 (intermediate vs low).

## Discussion

For the first time, this study validated the Halabi nomogram using real-world data. In this post-docetaxel second-line chemotherapy cohort, the Halabi nomogram had an AUC similar to that obtained in the internal validation. In addition, the two-risk group version confirmed the ability of the model to predict OS. However, this study did not sufficiently validate the three-risk group version.

The two-risk group stratification model differentiated the two curves throughout its entire trajectory. However, in the three-risk group stratification, the intermediate- and high-risk curves overlapped. This could be because of the low sample size and the small number of patients in the intermediate-risk group.

Notably, although the Halabi nomogram was developed to predict OS, in this dataset, the two-risk group version also differentiated PFS. However, further studies are required to prove any benefit in PFS in this stratification model.

Given the significant advances in therapeutic agents for the treatment of mCRPC, it is necessary to incorporate risk stratification into its management. Men who received cabazitaxel significantly improved their PFS (2.8 vs 1.4 months) and OS (15.1 vs 12.7 months) ^(6)^. In this study, the median OS was 9 months, compared to 15 months in the TROPIC trial and 14 months in the PROSELICA trial. In addition, the median PFS was similar to that in the TROPIC and PROSELICA trials.

Both the PROSELICA and TROPIC trials defined the presence of pain if the mean present pain intensity scale of McGill-Melzack was ≥1 (PROSELICA) or ≥ 2 (TROPIC), if the mean analgesic score derived from analgesic consumption was 10 points or more at baseline, or both ^(16), (17)^. However, we used another definition based on the daily consumption of narcotic or non-narcotic analgesics for pain derived from prostate cancer. Although these two definitions are not equivalent, in this study, we presented a pain rate at baseline (45%) similar to that of the TROPIC trial (45%) and lower than that of the PROSELICA trial (71%).

When comparing patients’ available background data, the median age and performance status were similar to those of the TROPIC and PROSELICA trials. The duration of hormonal use and hemoglobin level were similar to those of the TROPIC trial. However, this study included a slightly lower proportion of patients with progression within 6 months of docetaxel treatment than the proportion in the TROPIC and the PROSELICA trials (82%, 89%, and 86%, respectively). In addition, the median PSA level in our study was lower than those in the TROPIC and PROSELICA trials (59 ng/mL, 135 ng/mL, and 159 ng/mL, respectively). On the other hand, patients included in this study had a higher median alkaline phosphatase (288 IU/L vs 145 IU/L), measurable disease rate (70% vs 54%), and a slightly higher visceral disease rate (27% vs 25%) than those in the TROPIC trial. These differences, the small number of patients included, and the heterogeneity of the population could explain the differences in median OS. Moreover, mCRPC is a heterogeneous disease, and ethnic differences have been described ^(18), (19)^.

Although the Halabi model has proven useful using patient data from trials, it is a relatively complex nomogram that requires the use of nine parameters, which makes it impractical. This and the small number of patients undergoing second-line post-docetaxel treatment may be the reasons why the Halabi nomogram has not been widely used and validated.

The major limitation of this study was the small number of patients. In addition, the retrospective design could lead to bias. Although no distinction was made between death by disease and death by other causes, the low median OS of the cohort decreased the risk of bias. However, our study has provided external validation of a prognostic risk model in post-docetaxel patients with mCRPC receiving cabazitaxel. This was confirmed by the AUC in our cohort, similar to the validation of the model using data from the TROPIC and SPARC trials.

## Conclusion

The two-risk stratification version but not the three-risk group analysis confirmed the ability of the model to prognosticate survival. These results support the value of the Halabi nomogram in men receiving post-docetaxel second-line chemotherapy for mCRPC.

## Article Information

### Conflicts of Interest

None

### Acknowledgement

We would like to acknowledge the contributions of the following collaborators: Akito Yamaguchi at Harasanshin Hospital (Fukuoka), Takashi Dejima at Kyushu Central Hospital (Fukuoka), Satoshi Otsubo at Kitakyushu Municipal Medical Center (Kitakyushu), Akio Tsutsui at JCHO Kyushu Hospital (Kitakyushu), and Yoshifumi Hori at Miyazaki Prefectural Miyazaki Hospital (Miyazaki).

### Author Contributions

LB and MS participated in the design of the study and drafting of the manuscript and were responsible for completing the study. MS, MN, TT, NS, SH, TY, MH, KK, and ME were responsible for the clinical work of the study and recording of data. LB and MS reviewed the manuscript. All authors read and approved the final draft.

### Approval by Institutional Review Board (IRB)

Approval code issued by the institutional review board number 2019-230 by Kyushu University.

### Informed Consent

Informed consent was obtained from all individual participants included in the study.
